# Interaction between PSMD10 and GRP78 accelerates endoplasmic reticulum stress-mediated hepatic apoptosis induced by homocysteine

**DOI:** 10.1186/s13099-021-00455-z

**Published:** 2021-10-19

**Authors:** Kun Xiao, Shengchao Ma, Long Xu, Ning Ding, Hui Zhang, Lin Xie, Lingbo Xu, Yun Jiao, Huiping Zhang, Yideng Jiang

**Affiliations:** 1 NHC Key Laboratory of Metabolic Cardiovascular Diseases Research, Yinchuan, 750004 Ningxia People’s Republic of China; 2 Ningxia Key Laboratory of Vascular Injury and Repair Research, Yinchuan, 750004 Ningxia People’s Republic of China; 3 Luoyang Central Blood Bank, Luoyang, 471000 Henan People’s Republic of China; 4 School of Basic Medical Sciences, Ningxia Medical University, Yinchuan, 750004 Ningxia People’s Republic of China; 5 Department of Prenatal Diagnosis Center, General Hospital of Ningxia Medical University, Yinchuan, 750004 Ningxia People’s Republic of China; 6 Department of Physiology and Pathophysiology, School of Basic Medical Sciences, Ningxia Medical University, 1160 Sheng Li Street, Yinchuan, 750004 Ningxia Hui People’s Republic of China

**Keywords:** Apoptosis, Endoplasmic reticulum stress, Homocysteine, miR-212-5p, Proteasome 26S subunit non-ATPase 10

## Abstract

**Background:**

The liver plays an important role in production and metabolism of homocysteine (Hcy), which has been reported to be involved in liver injury. In our previous work, we confirm that Hcy can induce liver injury by activating endoplasmic reticulum (ER) stress. However, the underlying mechanisms remain largely unknown.

**Results:**

In present study, we established the Hcy-induced liver injury model by feeding *cbs*^+/−^ mice with high methionine diet, and found that a considerable mass of disordered arrangement of hepatocytes and enlarged space between hepatocytes were frequently occurred in the liver of *cbs*^+/−^ mice, accompanied with elevated expression levels of apoptosis-related proteins. In addition, Hcy could activate ER stress both in *cbs*^+/−^ mice and hepatocytes. Mechanistically, Hcy promoted the expression levels of proteasome 26S subunit non-ATPase 10 (PSMD10) in hepatocytes; and the expression of ER stress indicators and apoptosis-associated proteins were significantly suppressed when PSMD10 was silenced in hepatocytes under Hcy treatment. Moreover, bioinformatics analysis and luciferase reporter assay demonstrated that PSMD10 was a target gene of miR-212-5p. Consistently, miR-212-5p overexpression could inhibit ER stress-mediated apoptosis of hepatocytes under Hcy treatment. With the help of co-immunoprecipitation assay, we identified that the interaction between PSMD10 and GRP78 accelerated ER stress-mediated hepatic apoptosis induced by Hcy.

**Conclusions:**

Our findings indicate that miR-212-5p directly targets PSMD10 and subsequently activates ER stress to promote Hcy-induced apoptosis of hepatocytes. We propose that endogenous PSMD10 physically interacts with GRP78 to regulate ER stress. Our study may provide the therapeutic target for the liver injury induced by Hcy.

**Supplementary Information:**

The online version contains supplementary material available at 10.1186/s13099-021-00455-z.

## Background

Homocysteine (Hcy) is a non-essential sulfhydryl-containing amino acid that is derived from methionine metabolism. Liver is a major organ for Hcy metabolism [1]. Recently, epidemiological and experimental studies linked Hcy to a wide range of impaired liver function [2]. Meanwhile, hepatocyte apoptosis plays an important role in liver injury, which correlates with severity of liver disease and participates in the progress of hepatic fibrosis [3]. Therefore, exploring the molecular mechanisms of Hcy-induced hepatocyte apoptosis is crucial for the treatment of liver disease.

Many studies have indicated that Hcy promotes liver dysfunction through oxidative stress, endoplasmic reticulum (ER) stress activation and inflammatory response [4, 5]. ER stress often occurs in the cells demanding a high rate of protein synthesis and secretion as well as harmful stresses, including hypoxia, infection and exposure to a toxic substance. It is generally accepted that the unfolded protein reaction (UPR) signaling pathway plays an independent role in ER stress, of which may present opportunities for targeted therapies [6].

Meanwhile, the glucose-regulated protein 78 (GRP78) also plays a critical role in sensing ER stress, triggering UPR, and leading to cell apoptosis [7]. In addition, oncoprotein proteasome 26S subunit non-ATPase 10 (PSMD10) is reported to promote ER stress by up-regulating GRP78 and enhancing the activation of the UPR pathway in HCC cells [8]. Our previous study confirmed that dramatic increase in GRP78 expression induced by Hcy could accelerate ER stress-mediated liver injury [9]. However, the exact mechanism underlying the regulation of GRP78 in hepatocytes apoptosis induced by Hcy remains unclear.

MicroRNAs (miRNAs) are essential small endogenous non-coding RNAs that negatively regulate gene expression by binding to the 3′-untranslated regions (3′UTR) of target genes, and thereby take part in cell proliferation, differentiation and apoptosis [10, 11]. Among them, miR-212 located at chromosome 17p13.3 is up-regulated in cancers such as oral carcinoma and lung cancer, while it is down-regulated in colorectal cancer, prostate cancer and hepatocellular carcinoma. In addition, the circulating or liver miR-212 also plays a role in the biology of non-alcoholic fatty liver disease [12]. Although miR-212 has showed its important role in cancer and hepatic disease, whether it can mediate Hcy-induced liver injury is currently unknown.

In the present study, we explore the possible role of miR-212-5p in regulating ER stress-mediated hepatocytes apoptosis which is induced by Hcy treatment. The results show that miR-212-5p suppresses Hcy-induced ER stress in liver by targeting PSMD10 and thereby destroying the interaction between PSMD10 and GRP78. These findings demonstrate that PSMD10 is involved in the regulation of Hcy-induced liver injury, which may provide a novel PSMD10-based therapeutic target for liver injury.

## Results

### Homocysteine aggravates liver injury by promoting hepatocytes apoptosis

**Fig. 1:**
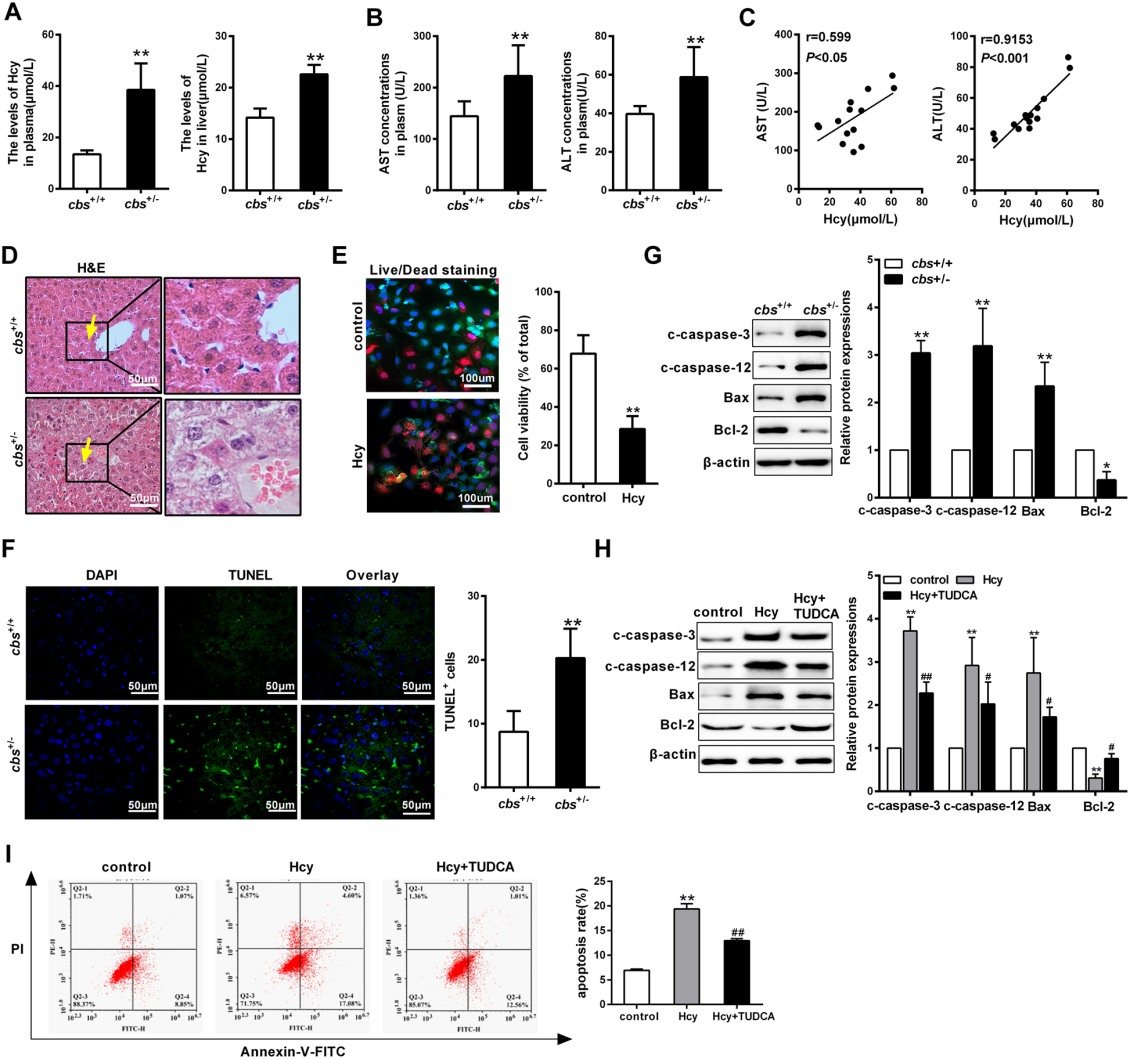
Homocysteine promotes apoptosis of hepatocytes leading to liver injury. **A** The levels of Hcy in plasma and liver were measured by automatic biochemical analysis and ELISA in *cbs*^+*/*+^ and *cbs*^+/−^ mice fed with high methionine diet (HMD) (n = 8/group). **B** The levels of serum aspartate aminotransferase (AST) and alanine transaminase (ALT) in *cbs*^+*/*+^ and *cbs*^+/−^ mice were measured by automatic biochemical analysis. **C** The correlation between serum Hcy levels and AST or ALT levels were evaluated by Pearson correlation analysis. **D** Representative photomicrographs of hematoxylin & eosin (HE) staining in liver sections from *cbs*^+/+^ and *cbs*^+/−^ mice (scale bars = 50 μm) **E** Representative immunofluorescence images of alive (green) and dead (red) hepatocytes after treatment with 100 μmol/L Hcy (scale bars = 100 μm). Nuclei were stained with DAPI (blue) **F** Apoptotic hepatocytes in the liver of *cbs*^+*/*+^ and *cbs*^+/−^ mice were assessed by TUNEL staining (scale bars = 50 μm). **G** Representative western blot and quantification of Bax, Bcl-2, cleaved caspase-3 and cleaved caspase-12 in the liver tissue of *cbs*^+/+^ and *cbs*^+/−^mice. **H** The protein levels of Bax, Bcl-2, cleaved caspase-3 and cleaved caspase-12 were detected by western blot in hepatocytes treated with 100 μmol/L Hcy or Hcy + TUDCA for 48 h. **I** Apoptosis rate of hepatocytes was measured by flow cytometry after cells were treated with 100 μmol/L Hcy or Hcy + TUDCA for 48 h. The apoptotic indices are expressed as the number of apoptotic cells/the total number of counted cells × 100%. All data are expressed as mean ± SD. *P < 0.05, **P < 0.001 versus *cbs*^+*/*+^ mice or control group (without treated with Hcy); ^#^*P* < 0.05, ^##^*P* < 0.01 versus Hcy group

To get a better insight into the possible mechanism of liver injury induced by Hcy, *cbs*^+/−^ mice were fed with high methionine diet (HMD) as described in experimental procedures. An elevation of Hcy levels in plasma and liver were significantly increased in *cbs*^+/−^ mice (Fig. 1A). In parallel to that, the serum concentrations of indicators for liver injury, such as aspartate aminotransferase (AST) and alanine transaminase (ALT), were increased in *cbs*^+/−^ mice, which were positively correlated with the levels of Hcy (Fig. 1B, C). Histologically, a considerable mass of disordered arrangement of hepatocytes, enlargement of space of hepatocytes were frequently identified in the liver of *cbs*^+/−^ mice (Fig. 1D). Moreover, Hcy prominently reduced cell viability in cultured hepatocytes (HL-7702) (Fig. 1E). These results suggest that Hcy exacerbates liver injury in *cbs*^+/−^ mice.

We then evaluated the hepatocytes apoptosis in Hcy-induced liver injury by using TUNEL staining. The result showed that the numbers of TUNEL-positive cells were increased in the liver of *cbs*^+/−^ mice compared to that of *cbs*^+/+^ mice (Fig. 1F). Next, the levels of apoptosis-related proteins including cleaved caspase-3, cleaved caspase-12, Bcl-2 associated X (Bax) and Bcl-2 were measured by western blotting. A remarkably increase of cleaved caspase-3, cleaved caspase-12 and Bcl-2 associated X (Bax) was observed in the liver of *cbs*^+/−^ mice, while bcl-2 expression level was reduced in *cbs*^+/−^ mice, compared with those in *cbs*^+/+^ mice (Fig. 1G). Meanwhile, the apoptosis ratio and the protein levels of cleaved caspase-3, cleaved caspase-12 and Bax were increased in hepatocytes treated with Hcy, whereas Bcl-2 expression was suppressed by Hcy in the cells (Fig. 1H). Furthermore, the TUDCA (a ERs-specific inhibitor) could counteract the effect of Hcy on apoptosis in hepatocytes (Fig. 1H and I). These results demonstrate that Hcy aggravates liver injury, and ER stress may play a critical role in the process of hepatocyte apoptosis induced by Hcy.

### Homocysteine induces ER stress through UPR signaling pathway in hepatocytes

**Fig. 2:**
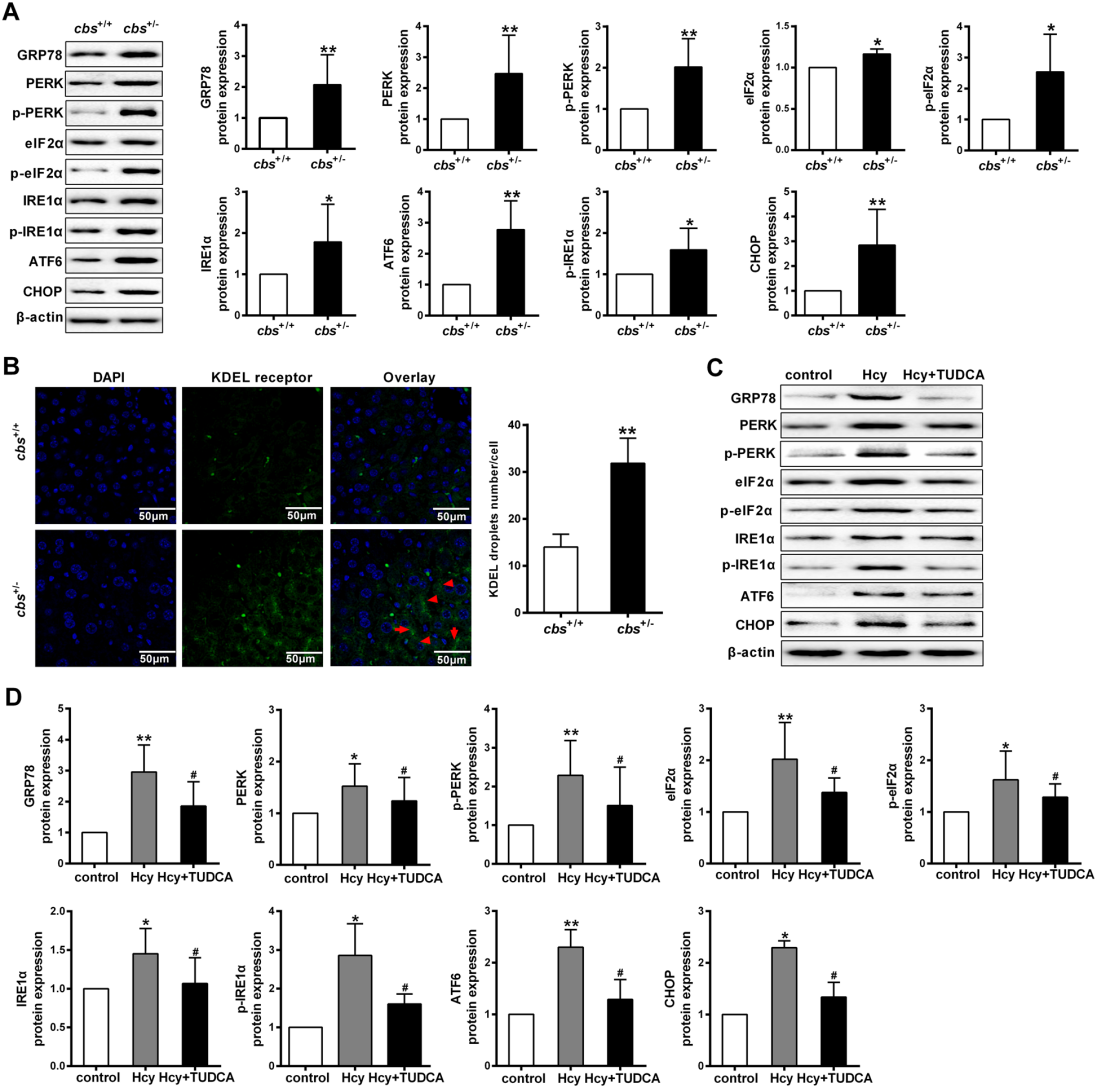
Homocysteine induces ER stress through UPR signaling pathway in hepatocytes. **A** The expression levels of ER stress signaling markers, including GRP78, ATF6, p-PERK, PERK, p-eIF2α, eIF2α, IRE1α, p-IRE1α and CHOP were measured by western blot in high methionine diet-fed *cbs*^+/+^ and *cbs*^+/−^ mice (n = 8/group). **B** Representative images for immunostaining analysis of KDEL receptor-positive cells in liver sections (scale bars = 20 μm). **C** and **D** The protein expression of ER stress signaling markers was assessed by western blot in hepatocytes treated with 100 μmol/L Hcy or Hcy + TUDCA for 48 h (n = 3/group). All data are expressed as mean ± SD. *P < 0.05, **P < 0.01 versus *cbs*^+*/*+^ mice or control group. ^#^*P* < 0.05, ^##^*P* < 0.01 versus Hcy group

ER stress can induce apoptosis through the UPR pathway, which is initiated by three transmembrane stress sensors of protein kinase RNA-like ER kinase (PERK), inositol-requiring protein 1α (IRE1α) and activating transcription factor 6 (ATF6) [13]. We then determined whether UPR pathway is involved in Hcy-induced ER stress of hepatocytes. The up-regulation of GRP78, PERK, p-PERK, eIF2α, p-eIF2α, IRE1α, p-IRE1α, ATF6 and C/EBP-homologous protein (CHOP) were observed in liver of *cbs*^+/−^ mice, compared to *cbs*^+/+^ mice (Fig. 2A). KDEL is a four amino acid sequence that allows retention of ER proteins in the ER [14]. The overall morphology of the hepatic lobule in the *cbs*^+/−^ mice exposure to high-methionine diet were changed, with decreased cell density and increased hepatocyte volume. In *cbs*^+/−^ mice, the overall intensity of KDEL immunostaining across the hepatic lobule and in hepatocytes was increased (Fig. 2B). Moreover, The expression levels of ER stress indicators were examined in hepatocytes treated with Hcy, where the expression levels of GRP78, CHOP, as well as PERK, IRE1α and ATF6 branches of UPR signaling, including p-eIF2α, p-PERK and p-IRE1α were markedly enhanced in the hepatocytes, which could be suppressed by TUDCA (Fig. 2C, D). Collectively, these results demonstrate that Hcy induces ER stress through UPR signaling pathway in hepatocytes.

### PSMD10 activates ER stress leading to apoptosis of hepatocytes induced by homocysteine

**Fig. 3:**
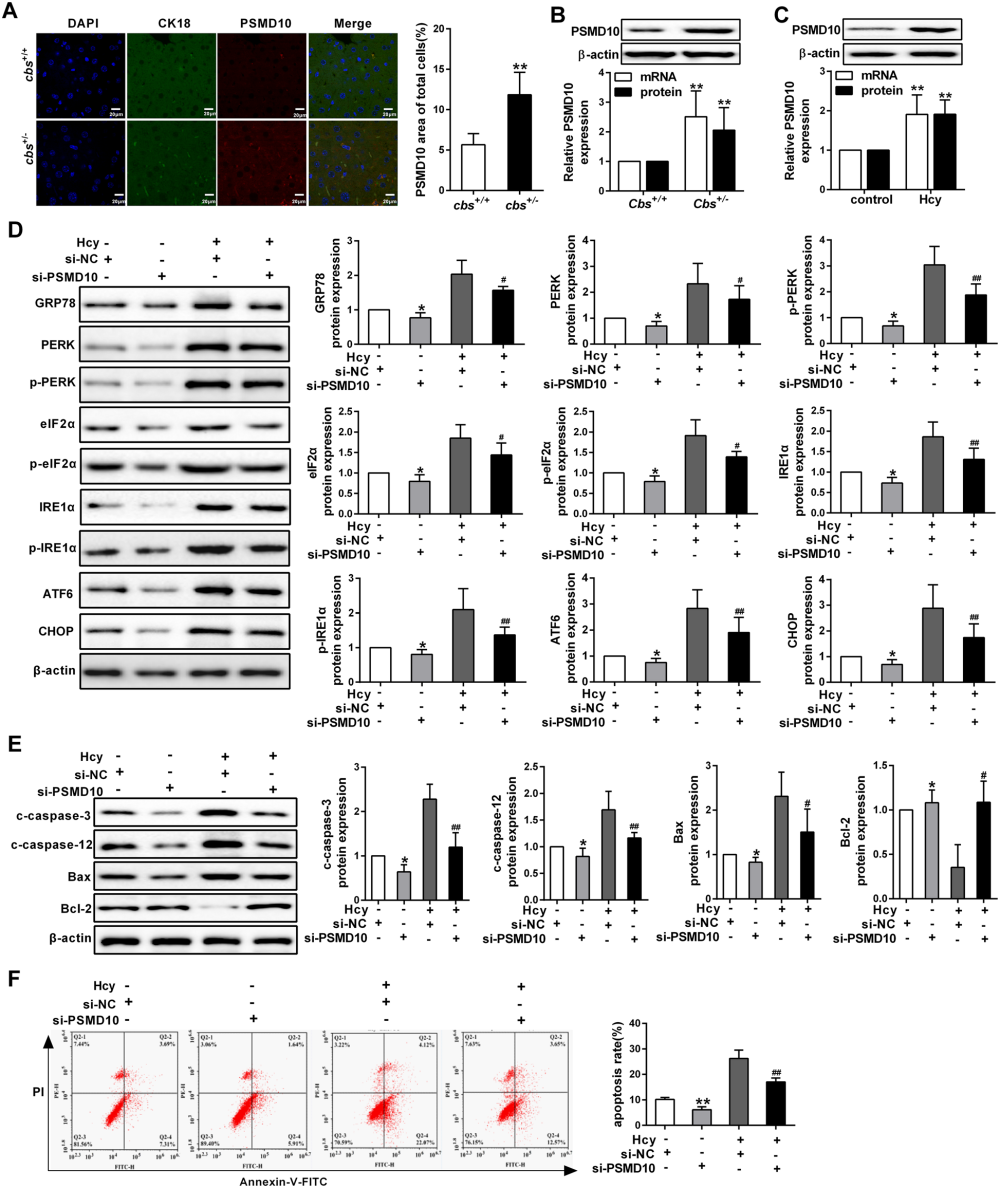
PSMD10 promotes hepatocytes apoptosis induced by homocysteine. **A** Co-immunofluorescent staining of PSMD10 (red) and CK18 (green, a positive marker for hepatocytes), nuclei were stained with DAPI (blue) (scale bars = 20 μm,). **B** The expression of PSMD10 was measured in the liver of *cbs*^+*/−*^mice by western blot and qRT-PCR. **C** Western blot and qRT-PCR were employed to detect the expression of PSMD10 in the hepatocytes which was treated with 100 μmol/L Hcy. **D**, **E** The protein expression of GRP78, p-PERK, PERK, p-eIF2α, eIF2α, IRE1α, p-IRE1α, CHOP, cleaved caspase-3, cleaved caspase-12, Bax and Bcl-2 was analyzed by western blot, after the hepatocytes were transfected with PSMD10 siRNA (si-PSMD10) and treated with 100 μmol/L Hcy. **F** Apoptosis of hepatocytes was analyzed by flow cytometry after the cells were transfected with si-PSMD10 in the presence of Hcy. All data are expressed as mean ± SD. *P < 0.05, **P < 0.01 versus *cbs*^+*/*+^, control or si-NC group. ^#^*P* < 0.05, ^##^*P* < 0.01 versus si-NC + homocysteine group

Previous study has demonstrated that PSMD10 is involved in cell apoptosis through enhancing the UPR signaling pathway [15]. To investigate the role of PSMD10 in hepatocytes apoptosis induced by Hcy, we performed co-immunofluorescent staining of CK18 (a positive marker of hepatocyte) and PSMD10 in the liver. Firstly, we observed PSMD10 positive cells predominantly co-localized with CK18 positive hepatocytes in the liver tissues of *cbs*^+/−^ mice (Fig. 3A). Besides, PSMD10 expression level was remarkably increased in the liver tissues of *cbs*^+/−^ mice, as well as in the Hcy-treated hepatocytes (Fig. 3B, C). To further explore the mechanisms by which PSMD10 regulates ER stress-mediated apoptosis, PSMD10 was overexpressed or silenced in hepatocyte by transfection with corresponding vectors (Additional file 1: Figure S1A, B). As expect, expression levels of ER stress-associated protein were significant decrease in HL-7702 hepatocytes treated with Hcy, such as GRP78, p-PERK, PERK, p-eIF2α, eIF2α, IRE1α, p-IRE1α and CHOP (Fig. 3D), and this trend was reversed when PSMD10 was overexpressed in the cells (Additional file 2: Figure S2A). Furthermore, the levels of cleaved caspase-3, cleaved caspase-12 and Bax were decreased after PSMD10 was silenced in HL-7702 hepatocytes under Hcy treatment, accompanied by an increased expression of bcl-2 (Fig. 3E and Additional file 2: Figure S2B). To determine the role of PSMD in Hcy-induced apoptosis in hepatocytes, the apoptosis ratio was analyzed by flow cytometry assay after siRNA against PSMD10 was transfected into hepatocytes under Hcy treatment. As shown in Fig. 3F, the apoptosis ratio of hepatocytes under Hcy treatment was markedly decreased when PSMD10 was silenced in the cells. Taken together, these data demonstrate that PSMD10 activates ER stress and promotes apoptosis of hepatocytes induced by Hcy.

### The interaction between PSMD10 and GRP78 facilitates ER stress-mediated apoptosis

**Fig. 4:**
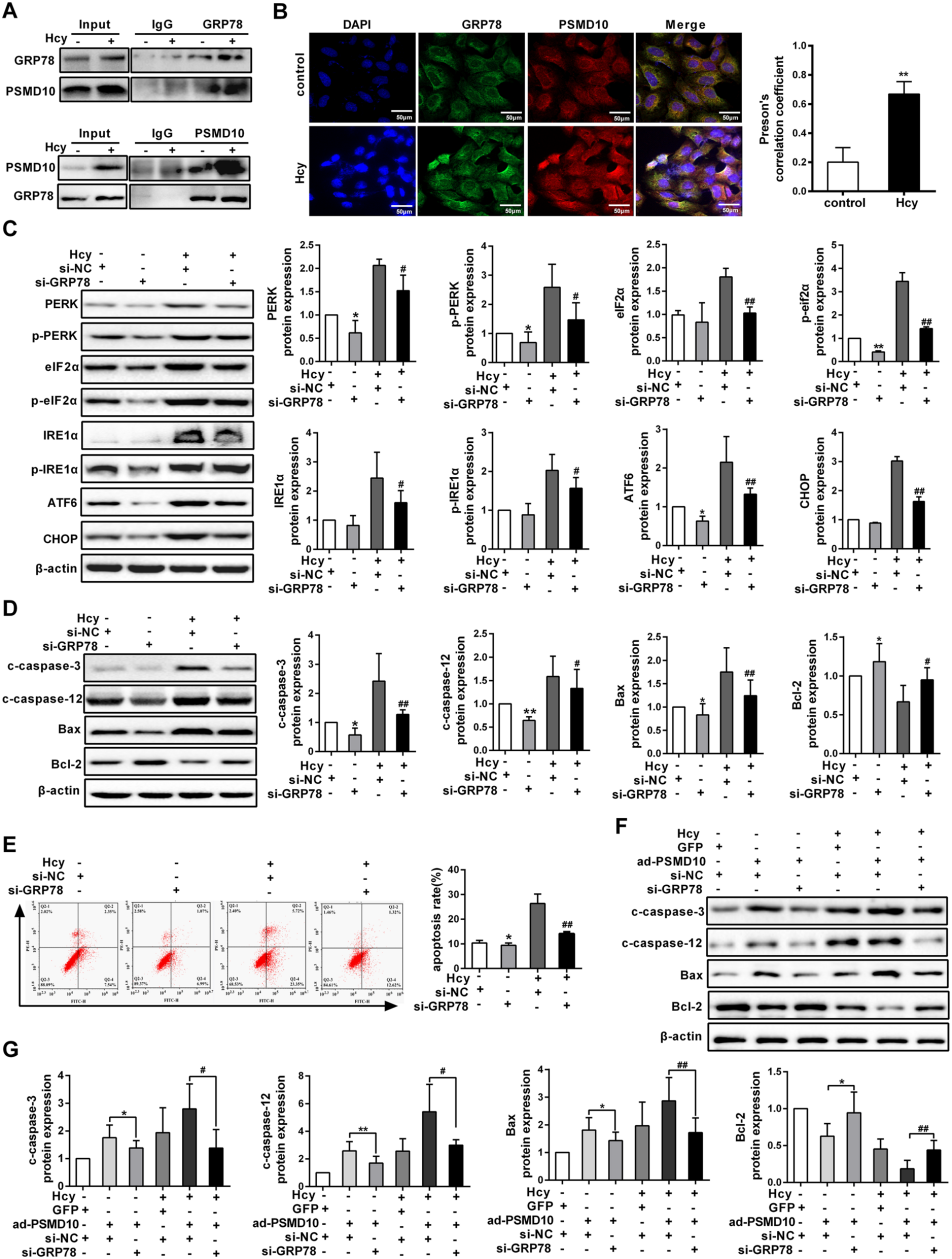
The cooperation between PSMD10 and GRP78 facilitates ER stress-mediated apoptosis induced by homocysteine. **A** The interaction between PSMD10 and GRP78 was confirmed by co-immunoprecipitation (Co-IP) in hepatocytes treated with Hcy. Upper panel: Co-IP using anti-GRP78 antibody; Lower panel: Co-IP using anti-PSMD10 antibody. **B** Co-immunofluorescent staining of GRP78 (green) and PSMD10 (red) in hepatocytes which was treated with 100 μmol/L Hcy (scale bars = 50 μm, 40×). Nuclei were stained with DAPI (blue). **C** The expression of p-PERK, PERK, p-eIF2α, eIF2α, IRE1α, p-IRE1α and CHOP in hepatocytes with or without Hcy treatment was detected by western blot, after the cells were transfected with si-NC or si-GRP78. **D** The expression of apoptosis-related markers was analyzed by western blot in hepatocytes after transfection with si-NC or si-GRP78 under Hcy treatment. **E** The apoptosis ratio of hepatocytes was measured by flow cytometry, after the cells were transfected with si-NC or si-GRP78 and treated with Hcy. **F**, **G** The protein levels of Bax, Bcl-2, cleaved caspase-3 and cleaved caspase-12 were assessed by western blot in the hepatocytes co-transduced with adenoviruses encoding PSMD10 (ad-PSMD10) and si-GRP78. All data are expressed as mean ± SD. *P < 0.05, **P < 0.01 versus si-NC group. ^#^*P* < 0.05, ^##^*P* < 0.01 versus si-NC + Hcy group or ad-PSMD10 + si-NC + Hcy group

GRP78, a biomarker of ER stress, is one of the best-characterized ER chaperone proteins [16]. Based on our previous findings, we next evaluated the mechanisms for PSMD10 activating ER stress. Firstly, co-immunoprecipitation (Co-IP) was employed to probe whether PSMD10 and GRP78 interact with each other. The result indicated that endogenous PSMD10 is able to co-immunoprecipitated GRP78, implying that PSMD10 physically interacted with GRP78 (Fig. 4A). Furthermore, we observed that Hcy promoted co-localization between endogenous GRP78 and PSMD10 in the cytoplasm of hepatocytes by co-immunofluorescent staining (Fig. 4B). To examine whether GRP78 is involved in activating ER stress and leading to apoptosis of hepatocyte induced by Hcy, we analyzed the expression of ER stress-associated protein by western blot, after GRP78 was silenced in hepatocytes under Hcy treatment. And we found a significant decrease in the expression of ER stress-associated protein, such as p-PERK, PERK, p-eIF2α, eIF2α, IRE1α, p-IRE1α, ATF6 and CHOP, which is accompanied by a synchronous decreased expression of apoptosis-associated protein, including cleaved caspase-3, cleaved caspase-12 and Bax (Additional file 3: Figure S3A, Fig. 4C, D). In addition, flow cytometry analysis also showed an obvious reduce of hepatocytes apoptosis after GRP78 knockdown in hepatocytes treated with Hcy (Fig. 4E). These results imply that GRP78 promotes ER stress-mediated apoptosis induced by Hcy. We further investigated the functional relationship between PSMD10 and GRP78 in vitro through co-transduction of ad-PSMD10 and si-GRP78 into hepatocytes, and found that up-regulation of PSMD10 apparently enhanced the expression of apoptosis-related proteins in hepatocytes treated with Hcy, which was reversed by knockdown of GRP78 in the cells (Fig. 4F, G). These data indicate a cooperative role of PSMD10 and GRP78 in facilitating ER stress-mediated apoptosis induced by Hcy.

### Homocysteine induces ER stress-mediated hepatocytes apoptosis by suppressing miR-212-5p

**Fig. 5:**
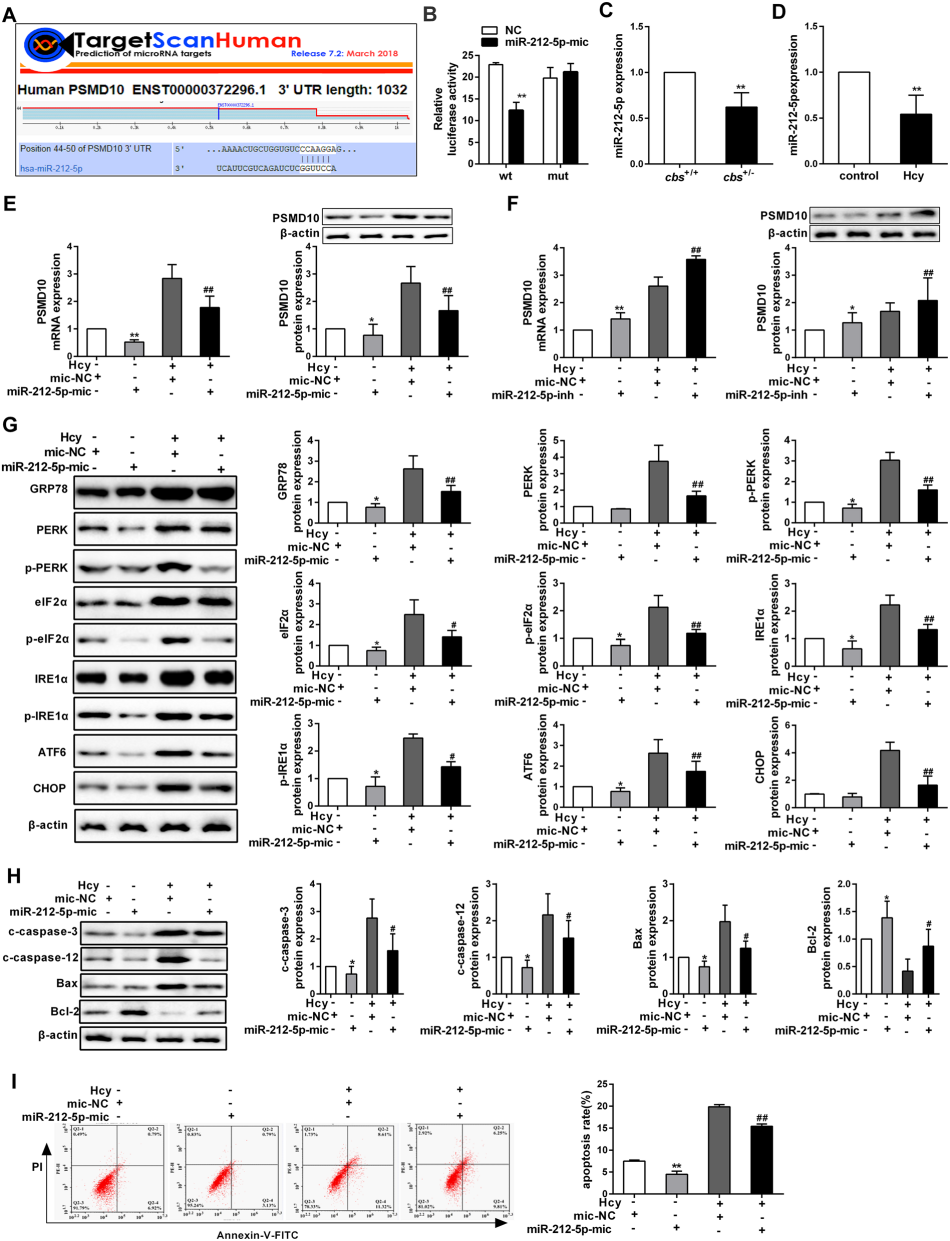
miR-212-5p regulates the apoptosis of hepatocytes induced by Hcy via targeting PSMD10. **A** Schematic representation of binding sites of miR-212-5p in the PSMD10 3′UTR analyzed by TargetScan database (http://www.targetscan.org/vert_72/). **B** Luciferase activity of pMIR-PSMD10-wt or pMIR-PSMD10-mut reporters in the presence of miR-212-5p mimics or negative control (NC) (n = 3). **C**, **D** The levels of miR-212-5p in *cbs*^+/−^ mice livers and the hepatocytes treated with 100 μmol/L Hcy were detected by qRT-PCR and normalized by U6. **E**, **F** PSMD10 expression was measured by qRT-PCR and western blot in hepatocytes after transfection with miR-212-5p mimics, miR-212-5p inhibitor or miR-neg control. **G**, **H** The expression levels of GRP78, p-PERK, PERK, p-eIF2α, eIF2α, CHOP, ATF6, cleaved caspase-3, cleaved caspase-12, Bcl-2 and Bax were detected by western blot in hepatocytes transfected with miR-212-5p mimic and treated with 100 μmol/L Hcy. **I** The apoptosis ratio of hepatocytes was analyzed by flow cytometry analyses after the cells were transfected with miR-212-5p mimic and treated with 100 μmol/L Hcy. All data are expressed as mean ± SD. ^*^P < 0.05, ^**^P < 0.01 versus *cbs*^+/+^, control, mic-NC or inh-NC group ^#^*P* < 0.05, ^##^*P* < 0.01 versus mic-NC + homocysteine or inh-NC + homocysteine group.

Since miRNAs are a class of important post-transcriptional regulators, we sought to investigate whether Hcy promotes apoptosis of hepatocytes through miRNAs targeting PSMD10. Based on the TargetScan database, a potential binding site of miR-212-5p at the 3′-UTR of PSMD10 was found (Fig. 5A). Luciferase reporter assay also illustrated that miR-212-5p mimic significantly inhibited the luciferase activity in hepatocytes transfected with reporter vectors containing wild type (wt) 3′-UTR of PSMD10 (Fig. 5B). These data suggest that miR-212-5p negatively regulates the expression of PSMD10 by directly binding to the putative binding sequences.

In order to further explore the role of miR-212-5p in apoptosis of hepatocytes induced by Hcy, we analyzed the effects of Hcy on the expression of miR-212-5p. As expected, miR-212-5p expression was suppressed in the liver of *cbs*^+/−^ mice and the Hcy-treated hepatocytes (Fig. 5C, D). Furthermore, down-regulation of PSMD10 was observed in hepatocytes transfected with miR-212-5p mimics, compared to negative control. On the contrary, PSMD10 expression was increased when the cells were transfected with miR-212-5p inhibitor (Additional file 4: Figure S4A, Fig. 5E, F). In addition, the expression levels of ER stress-associated proteins, such as p-IRE1α, p-PERK, p-eIF2α, IRE1α, PERK, eIF2α, ATF6 and GRP78, were suppressed in hepatocytes transfected with miR-212-5p mimic and treated with Hcy (Fig. 5G, Additional file 5: Figure S5A). Meanwhile, protein levels of cleaved caspase-3, cleaved caspase-12 and Bax were significantly decreased, while Bcl-2 was increased, when miR-212-5p mimics transfected into hepatocytes (Fig. 5H); and the trend is contrary to the results from cells transfection with miR-212-5p inhibitor (Additional file 5: Figure S5B). In line with the expression of apoptosis-associated proteins, the apoptosis ratio of hepatocytes was significantly decreased when miR-212-5p mimics transfected into cells and treated with Hcy, whereas miR-212-5p inhibitor had opposite effect (Fig. 5I and Additional file 5: Figure S5C). Taken together, these results indicate that Hcy activates ER stress-mediated hepatocytes apoptosis by down-regulating miR-212-5p and thereby enhancing the expression of its target gene, PSMD10.

## Discussion

Homocysteine (Hcy) in the liver is methylated to methionine by methionine synthase (requiring folate) and betaine-homocysteine methyltransferase, or converted to cystathionine by cystathionine synthase (CBS) [17]. Dysfunction of liver alters methionine metabolism, resulting in elevated Hcy that is released into the plasma; on the other hand, Hcy can influence the status of liver as well [18]. Our previous study proposed that ER stress might contribute to the pathogenic effects of Hcy on liver injury, while its molecular mechanism remains obscure [19]. In this study, we investigated the function of PSMD10 in liver injury caused by Hcy-induced ER stress, which is an alternative mechanism for the pro-apoptotic effect of Hcy. This was further confirmed by activation of PERK, p-PERK, IRE1α, p-IRE1Α and ATF6, the three major mediators of the ER stress response, as well as eIF2α and p-eIF2α. Activation of PERK causes phosphorylation of the eukaryotic translation initiation factor 2a and then inhibits protein synthesis. Activated IRE1 catalyzes the removal of a small intron from the XBP-1 mRNA, which triggers the induction of ER chaperones and other genes involved in ER-associated protein degradation [20, 21]. ATF6 and XBP-1 may combine to the ER stress response element and the UPR element, leading to GRP78 expression. Additionally, induction of CHOP, indicative of prolonged ER stress and pro-apoptotic signaling, further supported a Hcy-induced ER stress response linked to apoptosis [22]. GRP78 is the typical molecule that binds to ATF6, PERK, and IRE1 in normal condition [23]. When the cells are under stress, these proteins will be separated from GRP78 and activate downstream signal pathway to initiate UPR [24]. We found that Hcy remarkably up-regulates GRP78 both in vivo and in vitro, and GRP78 promotes ER stress-mediated apoptosis induced by Hcy, which is consistent with previous reports. Interestingly, the recent discovery that cannabidiol causes activated hepatic satellite cell death through a mechanism of ER stress-induced apoptosis [25], further confirms our results that Hcy promotes ER stress-mediated apoptosis to induce liver injury.

PSMD10 is involved in diverse biological processes, such as cell growth, proliferation, apoptosis and invasion, and contributes to oval cell-mediated liver regeneration and cell cycle progression [26]. In addition to normal biological functions, emerging evidences support that PSMD10 functions as an oncogene in many cancers including hepatocellular carcinomas, gliomas lung cancer, and colon, and so on [27]. Moreover, the abnormal expression of PSMD10 is related to the clinicopathological parameters of the disease, elucidating the important function of PSMD10 as a potential biomarker for diagnosis of different disease and a therapeutic target for disease treatment. Consistent over-expression of PSMD10 promotes tumor growth and inhibits apoptosis in hepatocellular carcinomas cells by enhancing the UPR and up-regulating GRP78 expression. As expected, we found that PSMD10 promotes ER stress-mediated hepatocytes apoptosis induced by Hcy, which agrees with previous report that PSMD10 augments CCl4-mediated chronic hepatic injury, inflammation and compensatory proliferation via the Rac1/JNK pathway [28]. However, what contradicts with us is that PSMD10 protects hepatocellular carcinoma cells from ER stress-induced apoptosis through the enhancement of UPR signaling. A recent study reported that PSMD10 interacts with the IL-1β/IRAK-1 inflammatory signaling pathway, and thereby induces the binding of the nuclear factor (NF-Y) complex to the PSMD10 promoter, facilitates the recruitment of the E1A-binding protein p300 and CREB-binding protein, and finally increases PSMD10 expression; in addition, PSMD10 promotes Rb phosphorylation and inactivation through interaction with cyclin-dependent kinase 4 and retinoblastoma protein (Rb), and activates the E2F transcription factor, leading to cell cycle progression [29]. Considering the interaction between PSMD10 and proteins, we also investigated if PSMD10 and GRP78 interact with each other. We firstly found that PSMD10 interacts with GRP78, leading to up-regulation of GRP78 and thereby acceleration of ER stress-mediated hepatic apoptosis induced by Hcy.

Additionally, PSMD10 could be regulated by miRNA at the posttranscriptional level. Recent report provided evidence that miR-605 significantly represses intrahepatic cholangiocarcinoma (ICC) cell proliferation and invasion by down-regulating PSMD10 through binding to the 3′UTR of PSMD10 [30]. Another study showed that ectopic expression of miR-214 inhibits myeloma cell growth and induces apoptosis by inhibiting PSMD10 [15]. Therefore, repressing PSMD10 activity may be a potential anticancer strategy. Previous studies have revealed many functions of miR-212-5p, such as tumor-promoting properties in NSCLC and proliferation-inhibition properties in gastric cancer [31, 32]. And many studies reported that miR-212-5p directly targets SIRT2 and prostaglandin endoperoxide synthase-2 to suppress the proliferation of colorectal cancer cells and protect against ferroptosis-mediated neuronal death [33, 34]. Consistent with these findings, our study indicated for the first time that miR-212-5p attenuates Hcy-induced hepatocytes apoptosis by targeting PSMD10.

## Conclusion

**Fig. 6:**
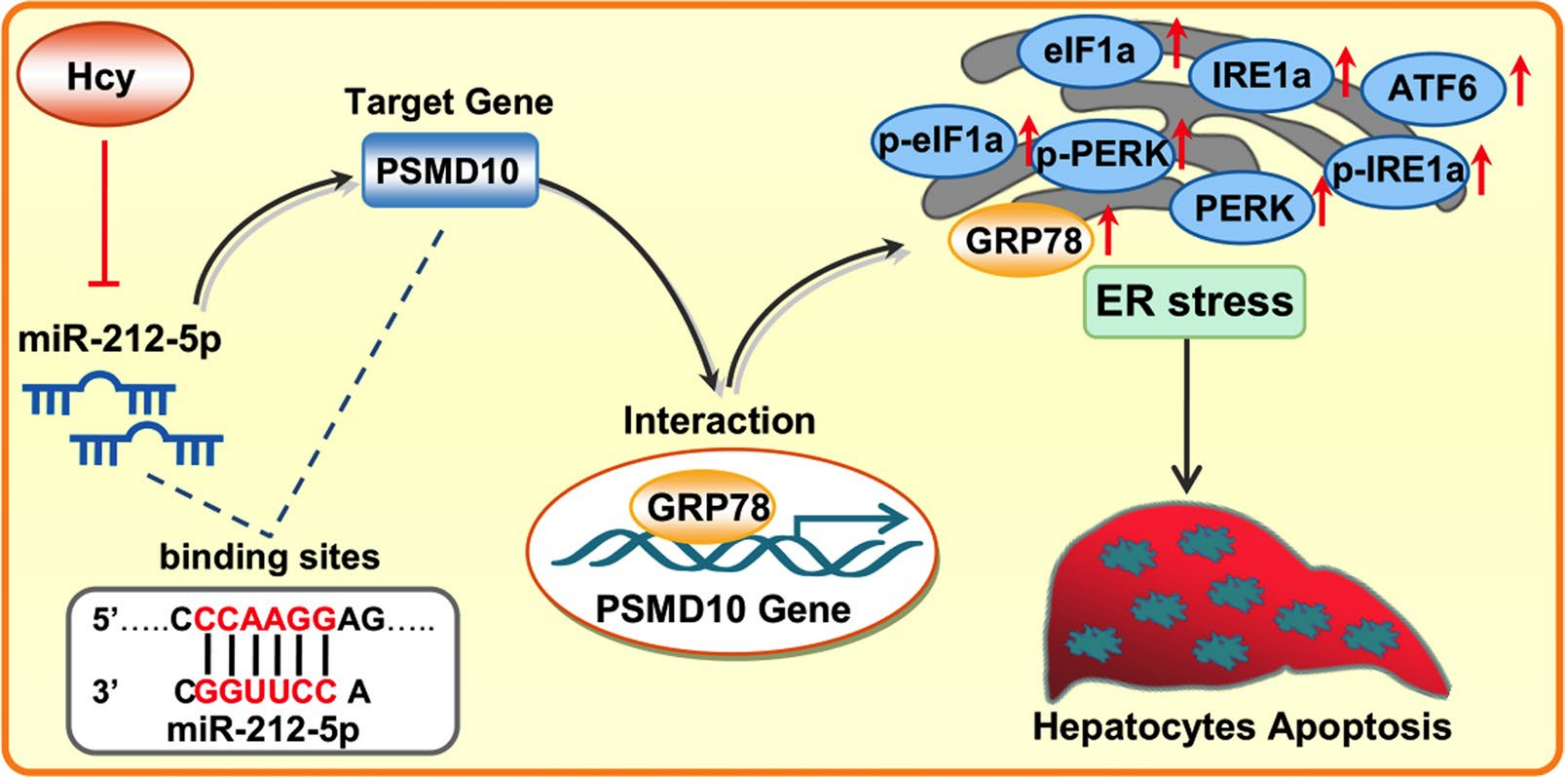
A proposed model of miRNA expression regulation in HHcy-induced liver injury. Under the condition of hyperhomocysteinemia (HHcy), down-regulated miR-212-5p specifically upregulates PSMD10 expression in hepatocytes. PSMD10 then activates ER stress through interacting with GRP78 and facilitates apoptosis of hepatocytes, by which Hcy induces liver injury

In summary, as shown in Fig. 6, we demonstrate that miR-212-5p is down-regulated and specifically regulates the expression of PSMD10 in Hcy-induced liver injury. Notably, the interaction between PSMD10 and GRP78 facilitates ER stress-mediated hepatocytes apoptosis induced by Hcy. These findings will give us a further insight into the potential of opening up novel therapeutic avenues for liver injury.

## Methods

### Animals

All animal experiments were approved and carried out in accordance with the Institutional Animal Care and Use Committee of the University of Ningxia Medical University. Eight to ten weeks old cystathionine beta-synthase (CBS) heterozygous knockout mice (*cbs*^+/−^) were obtained from Jackson Laboratory (Bar Harbor, ME) and maintained in the animal facility center at Ningxia Medical University. The mice were fed with regular diet plus 2.0% methionine chow and water ad libitum. Mice genotypes were determined by PCR.

### Cell culture, infection and transfection

The human HL-7702 hepatocyte cells were purchased from the BioWING Biotechnology (Shanghai, China) and cultured in RPMI-1640 medium (Thermo, USA) containing 10% fetal bovine serum (FBS) (Hyclone, USA) and 1% penicillin (Thermo, Waltham, MA, USA). Cells were incubated in a humidified incubator with 5% CO_2_ at 37 °C.

The cells were infected with adenoviruses encoding PSMD10 (Ad-PSMD10) when they were 80% confluent. The control cells were infected with Ad-GFP. Hepatocytes were transfected with non-silencing small interfering RNA (NC, 5′-UUCUCCGAACGUGUCACGUTT-3′), PSMD10 siRNA (5′-GCCGGGAUGAGAUUGUAAATT-3′), GRP78 siRNA (5′-GGGCAAAGAUGUCAGGA AATT-3′), miR-212-5p mimics (5′-ACCUUGGCUCUAGACUGCUUACU-3′) and miR-212-5p inhibitor (5′-AGUAAGCAGUCUAGAGCCAAGGU-3′) using Lipofectamine 2000 according to the manufacturer’s instructions (Invitrogen, Carlsbad, CA, USA).

### Liver tissue preparation and morphologic observation

All mice were fasted but supplied with water for 24 h followed by peritoneally injection with 3% pentobarbital sodium in a dose of 2 mL/kg. Abdominal cavity of anesthetic mouse was incised, and then inferior vena cava blood was collected using 10 mL syringe. After standing for 3 h at 4 °C, blood samples were centrifuged at 5000*g* for 15 min. Supernatant was stored at − 80 °C until future use. Liver was incised and two segments (1.0 cm × 1.0 cm × 0.3 cm) were cut from right lobe of the liver. Two liver segments were fixed in 10% neutral formalin and embedded in optimum cutting temperature compound (OCT). To observe morphologic changes in liver tissues, liver segments was HE stained.

### TUNEL assay

Liver tissues were stained using a TUNEL Staining Kit (Roche Inc., Basel, Switzerland) and the TUNEL-positive cells were symbolized by fluorescein-dUTP with dNTP according to the manufacturer’s protocol of the in-situ apoptosis Detection Kit (Roche Inc, Basel, Switzerland). Cells with nuclear condensation/fragmentation and apoptotic bodies in the absence of cytoplasmic TUNEL reactivity (green staining of nuclei) were considered as apoptotic cells. Cell nuclei was counterstained with DAPI and visualized by fluorescent microscopy.

### Western blot

Western blot analysis was performed as previously described [11]. Liver tissues and HL-7702 cells were lysed in a lysis buffer (KeyGEN, China) containing the protease inhibitor phenylmethanesulfonylfluoride (PMSF, KeyGEN, China) at 4 °C for 30 min followed by centrifugation to remove cell debris. Protein concentration was measured using BCA protein assay kit (Beyotime Institute of Biotechnology). The protein was separated by SDS-PAGE and transferred to PVDF membranes followed by examination with antibodies against PSMD10, cleaved caspase-3, cleaved caspase-12, Bax, Bcl-2, ATF6, p-PERK, PERK, eIF2α, p-eIF2α, IRE1α, p-IRE1α, CHOP and β-actin (all from Abcam Inc., Cambridge, MA, USA) respectively. Signal intensity was analyzed with Bio-Rad image analysis (Bio-Rad, Hercules, CA, USA).

### Real-time PCR (qRT-PCR)

Total RNA was isolated from livers or cultured cells using the miRNA isolation kit (Thermo Scientific, Waltham, MA, USA), and reverse transcribed using TaKaRa Master Mix (Dalian, China). Real-time PCR examination of PSMD10 and GAPDH were performed using Maxima SYBR Green/ROX qPCR Master Mix assay (Thermo Scientific, Waltham, MA, USA). The expression levels of mRNA and miRNA were normalized using GAPDH or U6 as a reference gene. qRT-PCR primer sequences are listed in Table 1.

**Table 1:** Primer sequences for qRT-PCR analyses

Gene	GenBank	Primer sequence, 5′ to 3′
GAPDH	NM_002814.4	F: GGTGAAGGTCGGTGTGAACG
		R: CTCGCTCCTGGAAGATGGTG
PSMD10	NM_001256799.3	F: GAACTGACCAGGACAGCAGAACTG
		R: AGCAGAAGCCGCAATATGAAGAGG
U6		GCTTCGGCAGCACATATACTAAAAT
miR-212-5p		ACCTTGGCTCTAGACTGCTTACTG
GRP78	NM_001163434.1	F:ATGATGAAGTTCACTGTGGTGG
		R:CTGATCGTTGGCTATGATCTCC
Si-PSMD10		F:CCAGAUGCUAAGGACCAUUTT
		R:AAUGGUCCUUAGCAUCUGGTT
Si-GRP78		F:GGCCACUAAUGGAGAUACUTT
		R:AGUAUCUCCAUUAGUGGCCTT

For miRNA analysis, the expression of miR-212-5p was evaluated with the TaqMan miRNA-assay (Applied Biosystems, Foster City, CA, USA). U6 was selected as an internal control. The primers for miR-212-5p were synthesized by Ribo Biotechnology (Guangzhou, China). The primer sequences were listed in Table 1. The universal reverse primer was provided in the Kit. The PCR cycling conditions were as follows: pre-denaturation at 95 °C for 2 min; 35 cycles of 95 °C for 15 s, 60 °C for 30 s, and 72 °C for 10 s; and a final extension at 72 °C for 2 min. The 2^−ΔΔCt^ method was utilized to quantify the relative expression of each gene.

### Immunofluorescence staining

The paraffin embedded mouse liver tissue sections were permeabilized in PBS containing 0.1% Triton X-100 for 5 min, and then incubated with blocking solution (5% goat serum in PBS) at room temperature for 30 min, followed by incubation with primary antibody (PSMD10-1:50, KDEL receptor-1:50, CK18-1:50, GRP78-1:50) overnight at 4 °C. After washing with PBS for three times, tissues were incubated with fluorescein conjugated secondary antibodies (goat anti-mouse or goat anti-rabbit) at RT for 1 h. After washing with PBS for three times, they were stained with DAPI for 5 min. Sections were then assessed using fluorescence microscopy (OLMPUS FV1000 confocal laser scanning microscope, Tokyo, Japan).

### Live/dead staining assay

Cell viability was monitored under laser scanning confocal microscope (LSM710) using cell viability Imaging Kit (Roche Diagnostics, 06432379001) according to the manufacturer’s instruction.

### Cell apoptosis

Hepatocytes apoptosis was detected using the FITC-Annexin V/PI staining kit. After Hcy treatment, the cells were washed with ice-cold PBS, and incubated with fluoresce in conjugated Annexin V and PI for 15 min, cell apoptosis was analyzed using a FACS flow cytometer equipped with the FACS talion data management system and Cell Quest software (Becton Dickinson, San Jose, CA, USA).

### Dual luciferase assays

Hepatocytes in 6-well plates were co-transfected with 200 ng of either pMIR-PSMD10-wt (5′-GAG AGTGGAAGAAGCAAAACTGCTGGTGTCCCAAGGAGCAAGTATTTACATTGAGAATAA-3′) or pMIR-PSMD10 mut (5′-GAGAGTGGAAGAA GCAAAACTGCTGGTGTCGGTTCCAGCAA GTATTTACATTGAGAATAA-3′) together with 100 nM of miR-212-5p mimics or non-target miRNA mimics using Lipofectamine 2000. In 48 h after transfection, firefly and renilla luciferase activities were measured using the Dual-Glo luciferase assay kit (Gibco Life Technologies).

### Co-immunoprecipitation assays

Cells were lysed in a lysis buffer containing protease inhibitor on ice. After centrifugation, the supernatant was incubated with an anti-PSMD10, anti-GRP78 or normal rabbit IgG respectively at 4 °C overnight followed by incubation with Dynabeads Protein G. Immunocomplex was separated by SDS-PAGE and proceeded for western blot analysis.

### Statistical analysis

Results are expressed as the mean ± SD from at least three independent experiments. The data were analyzed using one-way ANOVA and additional analysis using the Student Newman-Keuls test for multiple comparisons within treatment groups or *t*-test for two groups. *P* < 0.05 was considered to be statistically significant.

## Supplementary Information

**Additional file 1: Figure S1.** (A) The expression levels of PSDM10 were detected by qRT-PCR and western blot in hepatocytes, after the cells were transfected with three fragments of PSMD10 siRNAs (si-PSMD10) for 48 h. (B) qRT-PCR and western blot were used to determine PSMD10 expression levels in hepatocytes, after the PSMD10-encoding adenoviruses (ad-PSMD10) were transduced into hepatocytes for 48 h. All data are expressed as mean ± SD. *P < 0.05, **P < 0.01, ^#^*P* < 0.05. ^##^*P* < 0.01.

**Additional file 2: Figure S2.** (A) The expression of GRP78, p-PERK, PERK, p-eIF2α, eIF2α, IRE1α, p-IRE1α and CHOP in hepatocytes were examined by western blot, after the cells were transfected with adenoviruses encoding PSMD10 and treated with Hcy. (B) Western blot was employed to detect the expression of cleaved caspase-3, cleaved caspase-12, Bcl-2 and Bax protein expression in hepatocytes, after the cells were transfected with ad-PSMD10 and treated with Hcy. All data are expressed as mean ± SD. *P < 0.05, **P < 0.01, versus si-NC group. ^#^*P* < 0.05, ^##^*P* < 0.01 versus si-NC + Hcy group.

**Additional file 3: Figure S3.** Hepatocytes were transfected with siRNAs against GRP78 (the siRNAs fragments of GRP78-1,-4 and -8) or scrambled short hairpin RNA (si-NC), respectively. qRT-PCR and western blot were performed to verify the silence efficiency. All data are expressed as mean ± SD. *P < 0.05, **P < 0.01, versus si-NC group.

**Additional file 4: Figure S4**. (A) The expression of miR-212-5p in hepatocytes was determined by qRT-PCR and normalized by U6, after the cells were transfected with miR-212-5p mimics. (B) miR-212-5p in hepatocytes transfected with miR-212-5p inhibitor was quantified by qRT-PCR and normalized by U6. All data are expressed as mean ± SD. **P < 0.01, versus mic-NC or inh-NC.

**Additional file 5: Figure S5.** (A, B) The expression levels of GRP78, p-PERK, PERK, p-eIF2α, eIF2α, CHOP, ATF6, cleaved caspase-3, cleaved caspase-12, Bcl-2 and Bax were detected by western blot in hepatocytes, after the cells were transfected with miR-212-5p inhibitor and treated with 100 μmol/L Hcy. (C) The apoptosis ratio of hepatocytes was analyzed by flow cytometry analyses after the cells was transfected with miR-212-5p inhibitor and treated with 100 μmol/L Hcy. All data are expressed as mean ± SD. *P < 0.05, **P < 0.01, versus or inh-NC group ^#^*P* < 0.05, ^##^*P* < 0.01 versus inh-NC + Hcy group.

## Data Availability

Not applicable.

## Abbreviations

Hcy: Homocysteine
ER: Endoplasmic reticulum
UPR: Unfolded protein reaction
GRP78: Glucose-regulated protein 78
PSMD10: Proteasome 26S subunit non-ATPase 10
AST: Aspartate aminotransferase
ALT: Alanine transaminase
PERK: Protein kinase RNA-like ER kinase
ATF6: Activating transcription factor 6
CHOP: C/EBP-homologous protein
CBS: Cystathionine beta-synthase
Co-IP: Co-immunoprecipitation
