# Supplementary figures and images for: Interaction between PSMD10 and GRP78 accelerates endoplasmic reticulum stress-mediated hepatic apoptosis induced by homocysteine
Source: Gut Pathog. 2021 Oct 19;13:63. doi: 10.1186/s13099-021-00455-z (PMC8527788; doi:10.1186/s13099-021-00455-z)

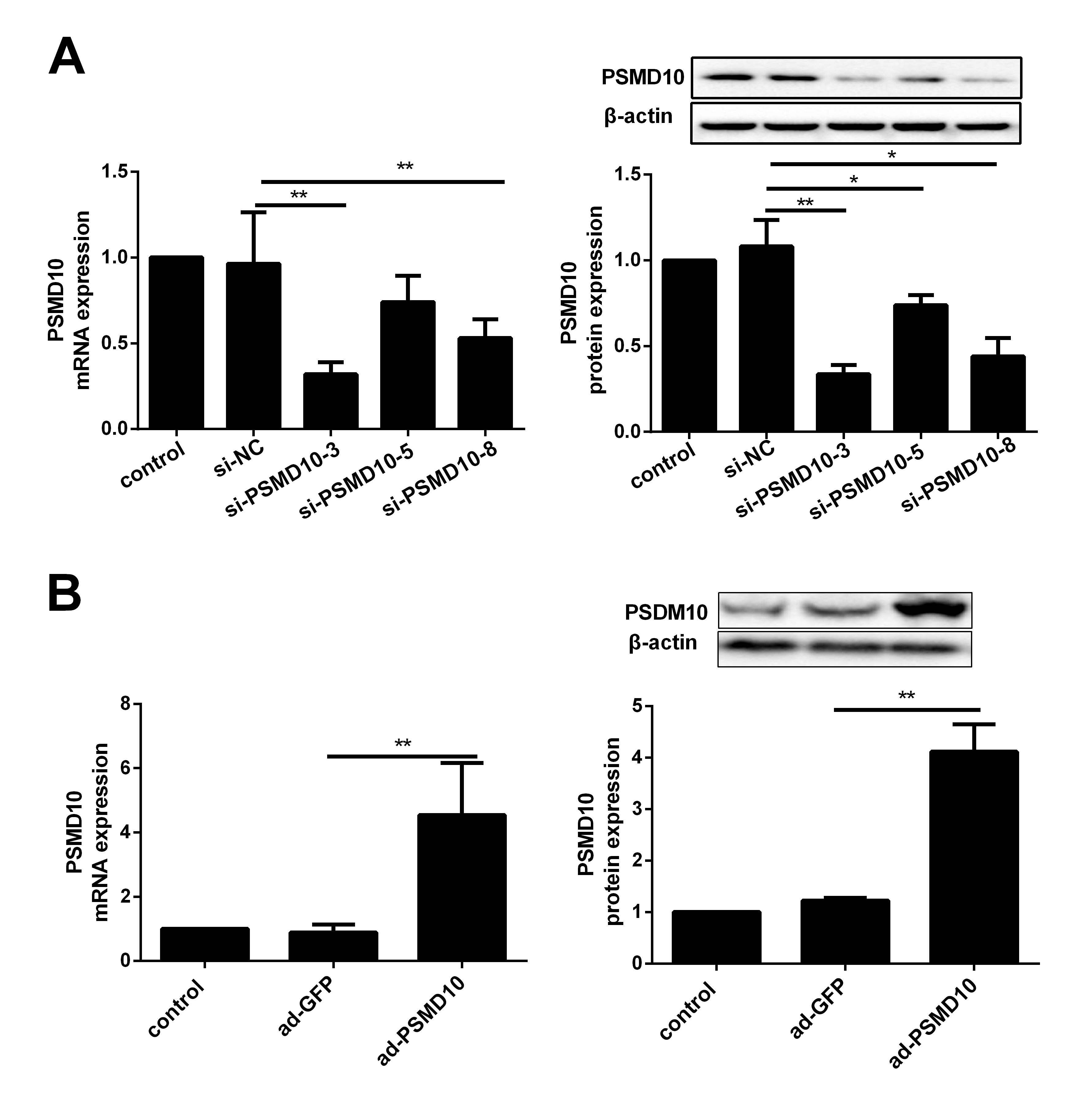

Supplement: Supplementary file 1 — Additional file 1: Figure S1. (A) The expression levels of PSDM10 were detected by qRT-PCR and western blot in hepatocytes, after the cells were transfected with three fragments of PSMD10 siRNAs (si-PSMD10) for 48 h. (B) qRT-PCR and western blot were used to determine PSMD10 expression levels in hepatocytes, after the PSMD10-encoding adenoviruses (ad-PSMD10) were transduced into hepatocytes for 48 h. All data are expressed as mean ± SD. *P < 0.05, **P < 0.01, #P < 0.05. ##P < 0.01. [file 13099_2021_455_MOESM1_ESM.tif]

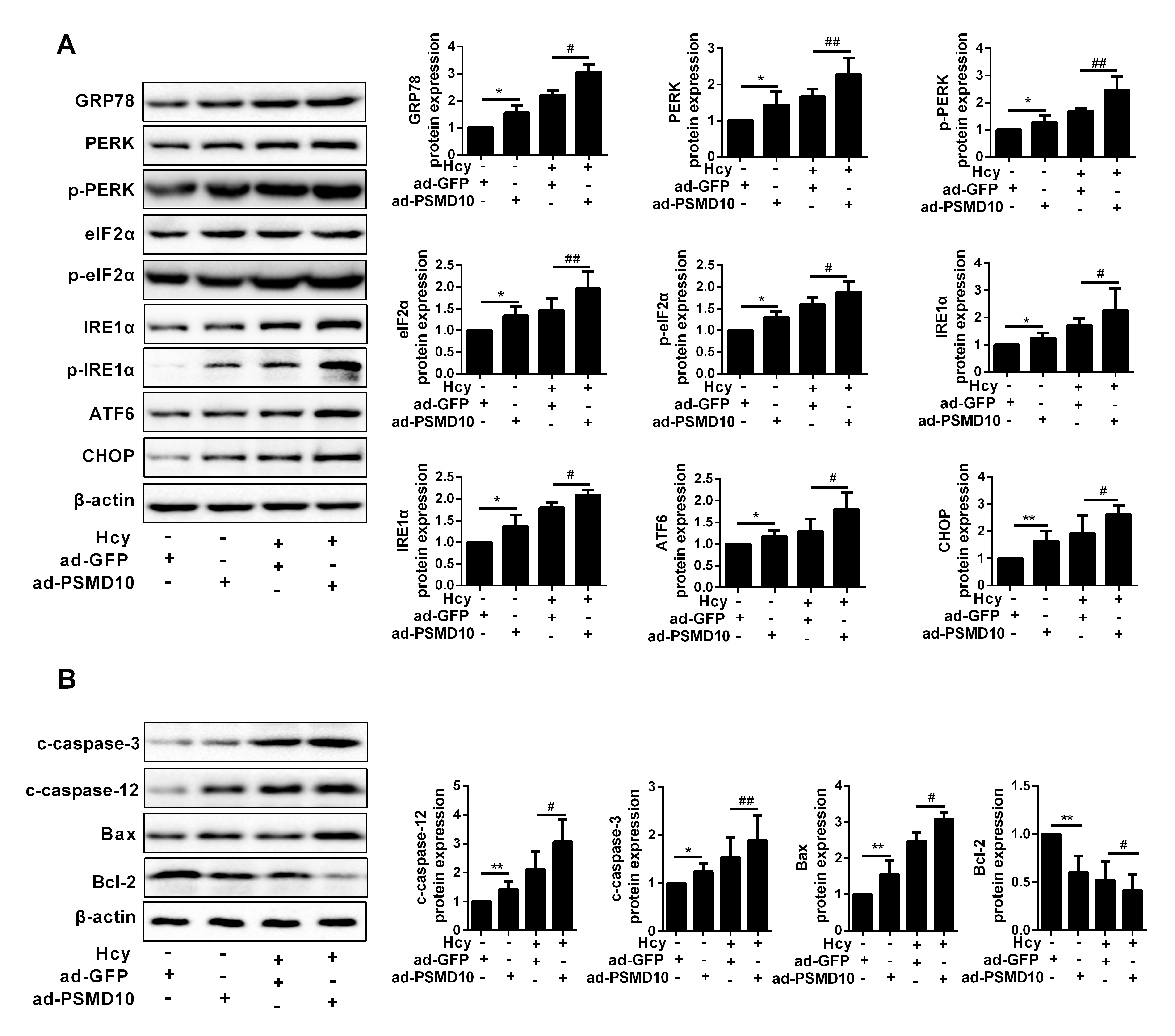

Supplement: Supplementary file 2 — Additional file 2: Figure S2. (A) The expression of GRP78, p-PERK, PERK, p-eIF2α, eIF2α, IRE1α, p-IRE1α and CHOP in hepatocytes were examined by western blot, after the cells were transfected with adenoviruses encoding PSMD10 and treated with Hcy. (B) Western blot was employed to detect the expression of cleaved caspase-3, cleaved caspase-12, Bcl-2 and Bax protein expression in hepatocytes, after the cells were transfected with ad-PSMD10 and treated with Hcy. All data are expressed as mean ± SD. *P < 0.05, **P < 0.01, versus si-NC group. #P < 0.05, ##P < 0.01 versus si-NC + Hcy group. [file 13099_2021_455_MOESM2_ESM.tif]

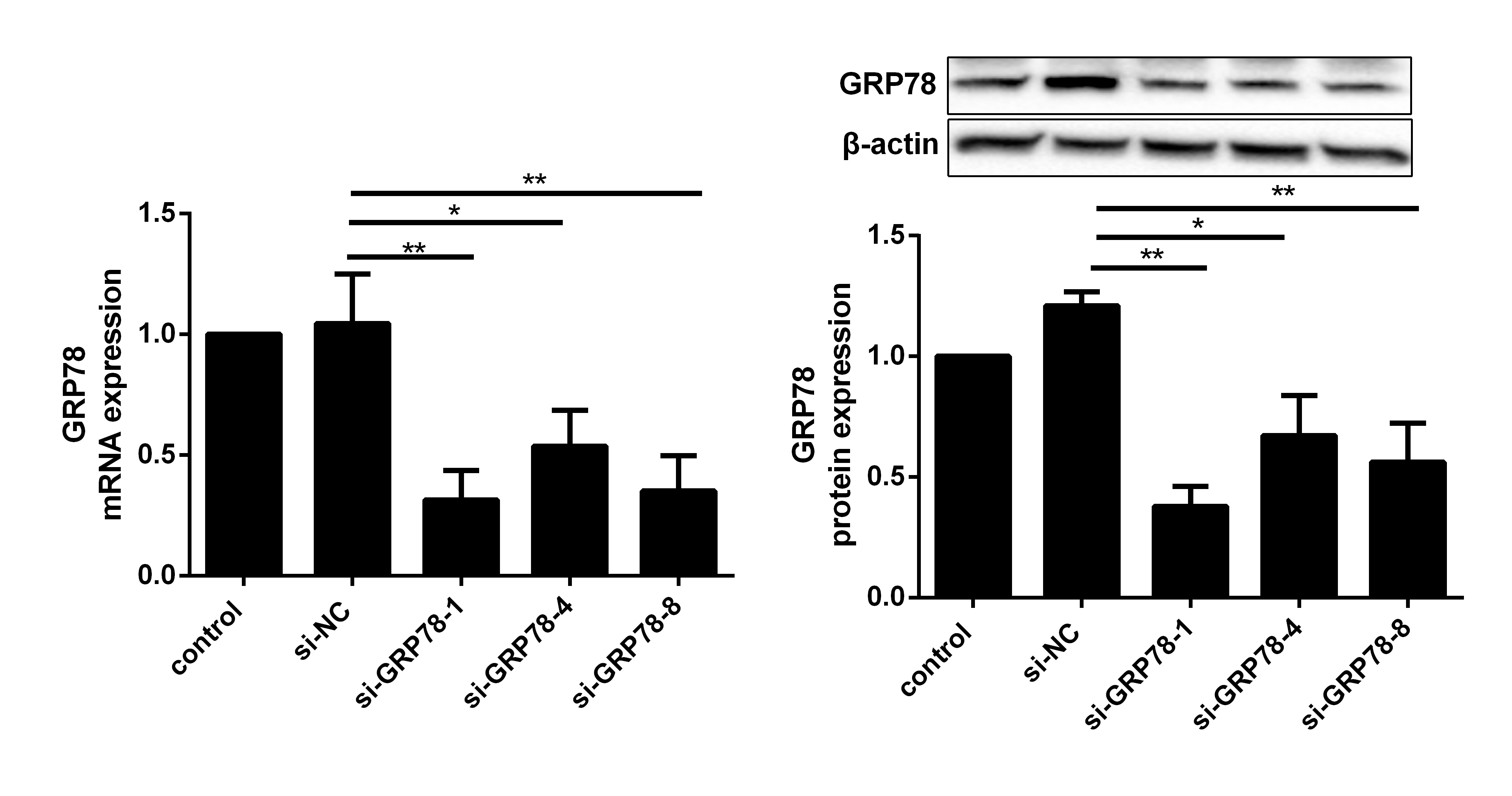

Supplement: Supplementary file 3 — Additional file 3: Figure S3. Hepatocytes were transfected with siRNAs against GRP78 (the siRNAs fragments of GRP78-1,-4 and -8) or scrambled short hairpin RNA (si-NC), respectively. qRT-PCR and western blot were performed to verify the silence efficiency. All data are expressed as mean ± SD. *P < 0.05, **P < 0.01, versus si-NC group. [file 13099_2021_455_MOESM3_ESM.tif]

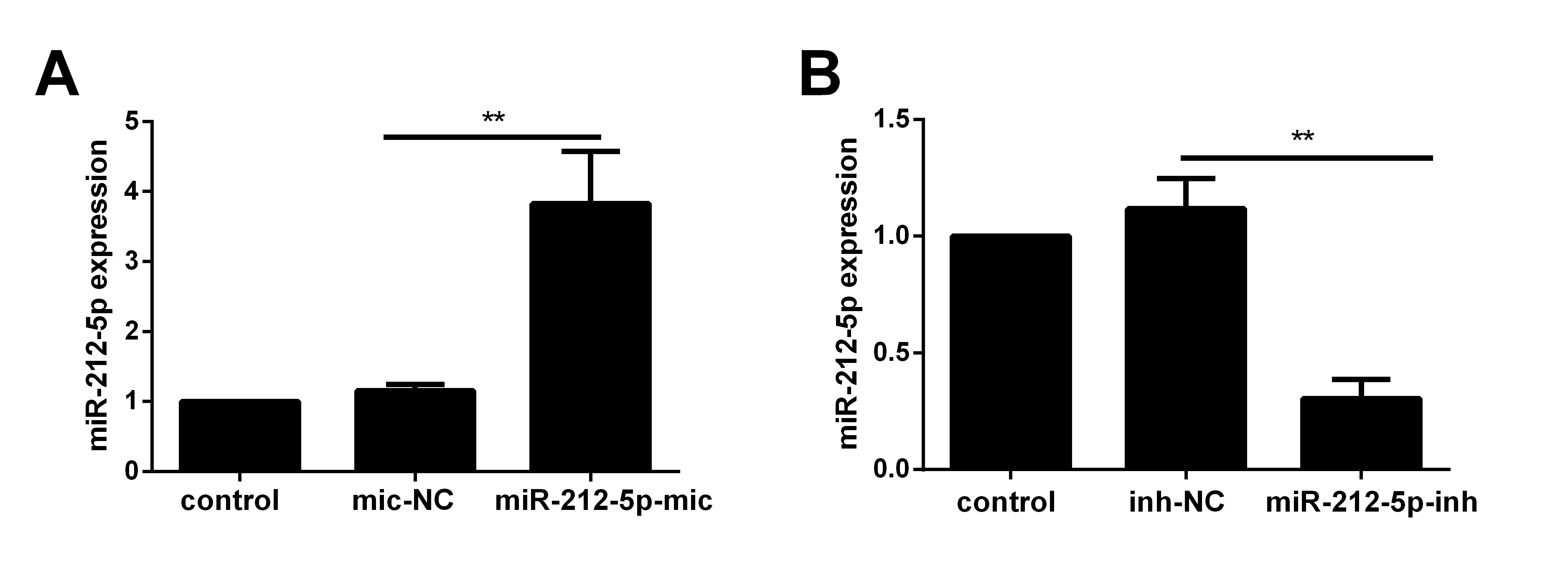

Supplement: Supplementary file 4 — Additional file 4: Figure S4. (A) The expression of miR-212-5p in hepatocytes was determined by qRT-PCR and normalized by U6, after the cells were transfected with miR-212-5p mimics. (B) miR-212-5p in hepatocytes transfected with miR-212-5p inhibitor was quantified by qRT-PCR and normalized by U6. All data are expressed as mean ± SD. **P < 0.01, versus mic-NC or inh-NC. [file 13099_2021_455_MOESM4_ESM.tif]

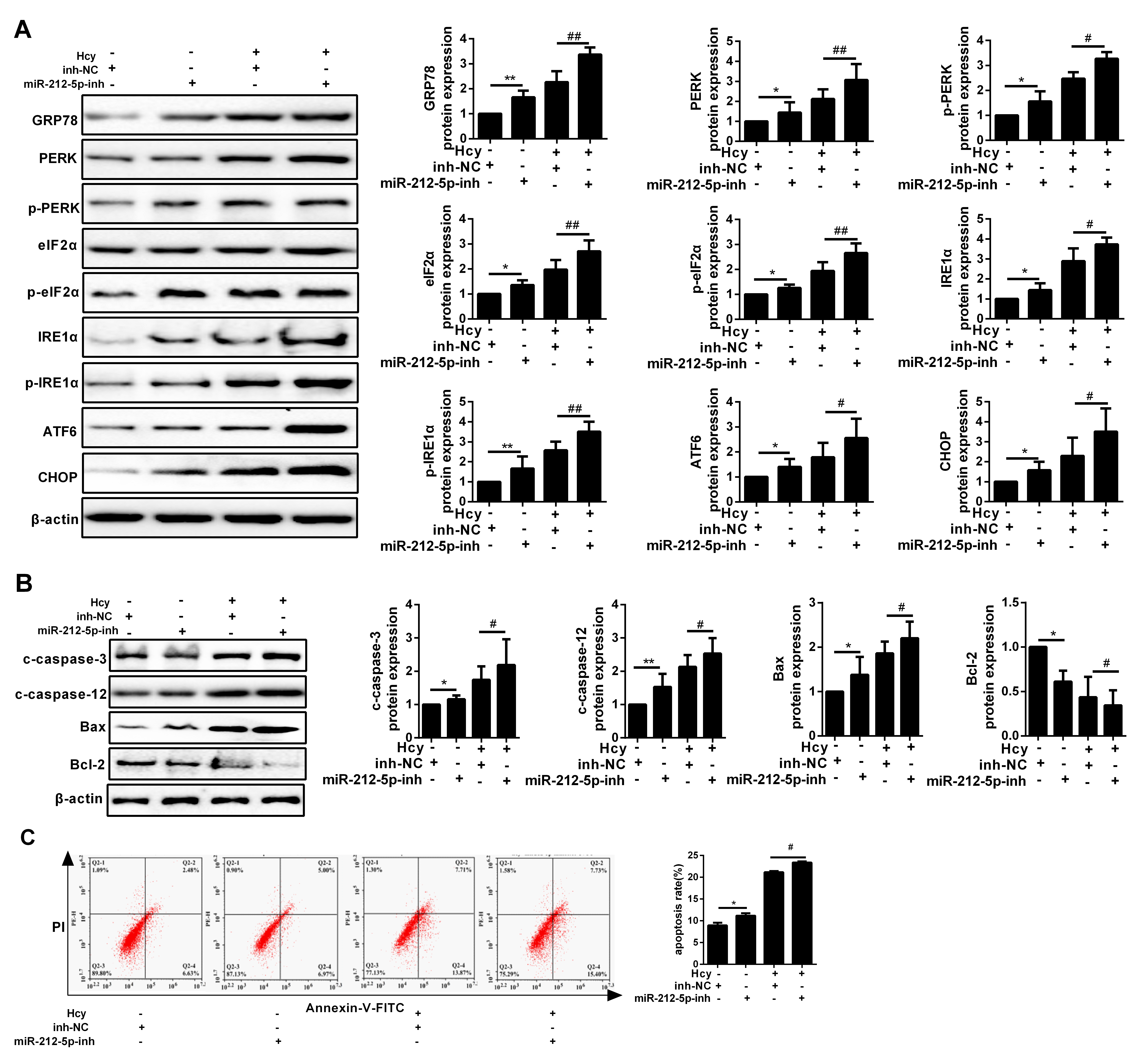

Supplement: Supplementary file 5 — Additional file 5: Figure S5. (A, B) The expression levels of GRP78, p-PERK, PERK, p-eIF2α, eIF2α, CHOP, ATF6, cleaved caspase-3, cleaved caspase-12, Bcl-2 and Bax were detected by western blot in hepatocytes, after the cells were transfected with miR-212-5p inhibitor and treated with 100 μmol/L Hcy. (C) The apoptosis ratio of hepatocytes was analyzed by flow cytometry analyses after the cells was transfected with miR-212-5p inhibitor and treated with 100 μmol/L Hcy. All data are expressed as mean ± SD. *P < 0.05, **P < 0.01, versus or inh-NC group #P < 0.05, ##P < 0.01 versus inh-NC + Hcy group. [file 13099_2021_455_MOESM5_ESM.tif]
